# *catena*-Poly[[cobalt(II)-μ-aqua-μ-propano­ato-κ^2^ *O*:*O*′-μ-propano­ato-κ^2^ *O*:*O*] monohydrate]

**DOI:** 10.1107/S1600536810043540

**Published:** 2010-10-31

**Authors:** A. I. Fischer, V. V. Gurzhiy, A. N. Belyaev

**Affiliations:** a St Petersburg State Institute of Technology, Moskovsky Prospekt 26, 190013 St Petersburg, Russian Federation; b St Petersburg State University, Universitetskaya Naberezhnaya 7/9, 199034 St Petersburg, Russian Federation

## Abstract

The title compound, {[Co(C_2_H_5_COO)_2_(H_2_O)]·H_2_O}_*n*_, was synthesized by the reaction of cobalt(II) carbonate hydrate with aqueous propionic acid. The structure consists of polymeric infinite linear chains with composition [Co(C_2_H_5_COO)_4/2_(H_2_O)_2/2_]_∞_ running along [010]. The chains are formed by Co^2+^ ions linked with bridging propionate groups and water mol­ecules, with a Co⋯Co distance along the chains of 3.2587 (9) Å. The Co^2+^ ion is six-coordinated in a strongly distorted octa­hedral geometry. The chains are connected to each other by a network of O—H⋯O hydrogen bonds involving solvent water mol­ecules.

## Related literature

For the related cobalt(II) acetate dihydrate, see: Jiao *et al.* (2000 ▶). For the structure of a hydrated cobalt(II) acetate which has been isolated in similar conditions, see: Sobolev *et al.* (2003 ▶). For properties and applications of cobalt carboxyl­ates, see: Eremenko *et al.* (2009 ▶); Gates (1992 ▶); Parshall & Ittel (1992 ▶); Partenheimer (1995 ▶).
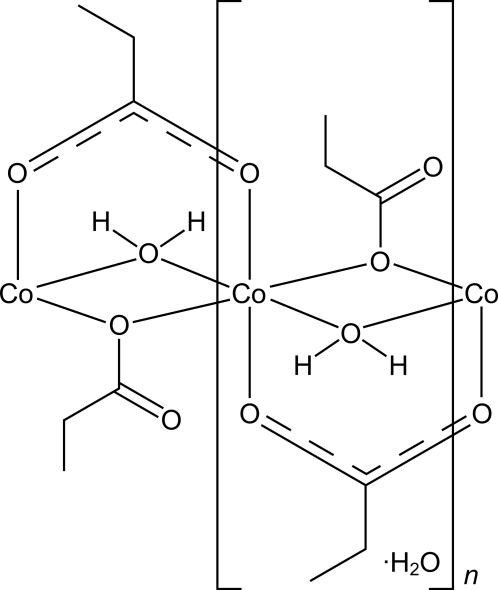


## Experimental

### 

#### Crystal data

[Co(C_3_H_5_O_2_)_2_(H_2_O)]·H_2_O
*M* *_r_* = 241.10
Monoclinic, 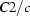

*a* = 13.997 (4) Å
*b* = 6.4987 (18) Å
*c* = 21.440 (6) Å
β = 103.216 (5)°
*V* = 1898.6 (9) Å^3^
*Z* = 8
Mo *K*α radiation
μ = 1.81 mm^−1^
*T* = 170 K
0.5 × 0.3 × 0.2 mm

#### Data collection

Bruker APEXII CCD diffractometer
Absorption correction: multi-scan (*SADABS*; Bruker, 2007 ▶) *T*_min_ = 0.276, *T*_max_ = 0.332
11986 measured reflections
2762 independent reflections
2542 reflections with *I* > 2σ(*I*)
*R*_int_ = 0.032

#### Refinement

*R*[*F* ^2^ > 2σ(*F* ^2^)] = 0.026
*wR*(*F* ^2^) = 0.067
*S* = 1.09
2762 reflections
130 parameters
5 restraints
H atoms treated by a mixture of independent and constrained refinement
Δρ_max_ = 0.41 e Å^−3^
Δρ_min_ = −0.57 e Å^−3^

### 

Data collection: *APEX2* (Bruker, 2009 ▶); cell refinement: *SAINT* (Bruker, 2009 ▶); data reduction: *SAINT*; program(s) used to solve structure: *SHELXS97* (Sheldrick, 2008 ▶); program(s) used to refine structure: *SHELXL97* (Sheldrick, 2008 ▶); molecular graphics: *DIAMOND* (Brandenburg, 2008 ▶); software used to prepare material for publication: *publCIF* (Westrip, 2010 ▶).

## Supplementary Material

Crystal structure: contains datablocks global, I. DOI: 10.1107/S1600536810043540/fj2358sup1.cif

Structure factors: contains datablocks I. DOI: 10.1107/S1600536810043540/fj2358Isup2.hkl

Additional supplementary materials:  crystallographic information; 3D view; checkCIF report

## Figures and Tables

**Table 1:** Hydrogen-bond geometry (Å, °)

*D*—H⋯*A*	*D*—H	H⋯*A*	*D*⋯*A*	*D*—H⋯*A*
O1—H11⋯O6^i^	0.92 (3)	1.72 (3)	2.620 (2)	163 (3)
O1—H12⋯O5	0.89 (3)	1.78 (3)	2.660 (2)	171 (3)
O5—H51⋯O4^ii^	0.92 (3)	1.90 (3)	2.794 (2)	163 (3)
O5—H52⋯O2^iii^	0.87 (3)	1.91 (3)	2.773 (2)	174 (3)

Symmetry codes: (i) 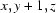; (ii) 
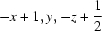
; (iii) 
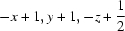
.

**Fig. 1.**
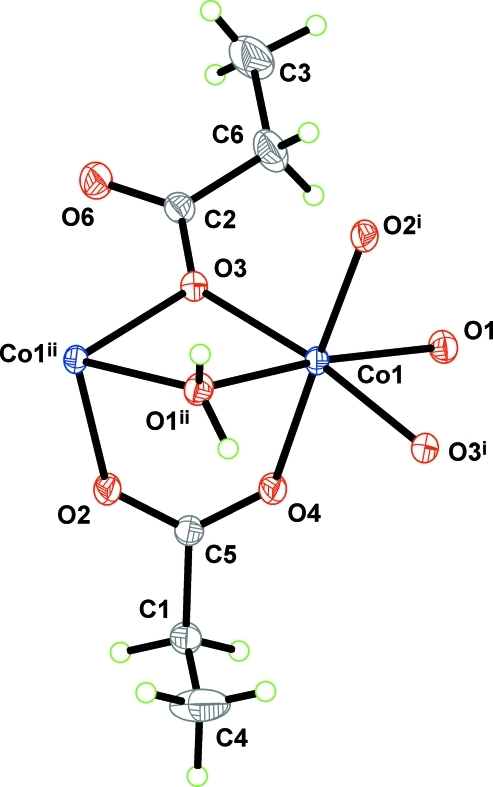
[Figure 1. The coordinated mode and linkage of the complex (I). Displacement ellipsoids of non-H atoms are drawn at the 40% probability level. Hydrogen atoms are drawn as spheres of arbitrary radius. Symmetry codes: i = –x + 1/2, y + 1/2,–z + 1/2; ii = –x + 1/2, y–1/2, –z + 1/2.]

**Fig. 2.**
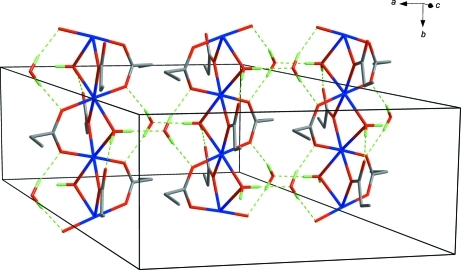
[Figure 2. Sticks representation of the crystal structure of (I) showing hydrogen bonds as green dashed lines. Only the H atoms involved in hydrogen bonds are shown. Co atoms are blue, O atoms are red, C atoms are grey, and selected H atoms are green colored.]

## supplementary crystallographic information

### Comment

Cobalt carboxylates are of great importance because of their application in homogeneous oxidation catalysis (Gates, 1992; Parshall & Ittel, 1992; Partenheimer, 1995), and their interesting magnetic properties (Eremenko *et al.*, 2009). Carboxylate ligands coordinated to transition metal ions can adopt different binding modes and form a great number of various cage complexes and a variety of different one-dimensional, two-dimensional and three-dimensional structures.

The title compound, [Co(C_2_H_5_COO)_2_(H_2_O)]*_n_*.*n*H_2_O (I), actually named as cobalt(II) propionate dihydrate, is attracting our attention as the starting substance for the synthesis of mixed-valence cobalt carboxylates. This salt was synthesized and its crystal structure is reported herein.

The crystal structure of (I) contains one symmetrically independent Co^2+^ cation coordinated to four O atoms of four bridging propionates and two O atoms of bridging water molecules in a strongly distorted octahedral coordination (Fig. 1). The *cis-*angles about the Co atom range from 77.35 (4) to 109.40 (5)°, the Co—O bond length ranges from 2.0406 (12) to 2.2460 (12) Å; this is in agreement with the angles and the distances in isostructural cobalt(II) acetate dihydrate (Jiao *et al.*, 2000). The structure of (I) consists of polymeric infinite linear chains with composition _∞_[Co(C_2_H_5_COO)_4/2_(H_2_O)_2/2_] running along [010]. The Co···Co separation is 3.2587 (9) Å. The bridging carboxylate groups adopt two coordination modes, monodentate and *syn-syn* bidentate. The bidentate carboxylate group has C—O bonds of equal length, 1.267 (2) Å, whereas monodentate carboxylate group has different C—O bond lengths, 1.235 (2) and 1.305 (2) Å. The chains are connected to each other by a network of hydrogen bonds to solvate water molecules (Fig. 2).

It is rather interesting that the use of acetic acid instead of propionic acid in the synthesis of (I) result in the formation of monomeric cobalt(II) acetate tetrahydrate (Sobolev *et al.*, 2003), and the polymeric cobalt(II) acetate dihydrate is formed by recrystallization of cobalt(II) acetate tetrahydrate from acetic acid (Jiao *et al.*, 2000).

### Experimental

To a solution of propionic acid (7.4 g, 100 mmol) in water (15 ml), an excess of fresh cobalt(II) carbonate hydrate, CoCO_3_.*x*H_2_O, (8.0 g, approximately 60 mmol) was added. The reaction mixture was stirred for 8 h at room temperature, followed by filtrating the unreacted CoCO_3_.*x*H_2_O out. The filtrate was allowed to stand at room temperature for slow evaporation. The red single crystals of (I) suitable for X-ray diffraction studies were obtained after several days. Yield 85%.

### Refinement

For a single-crystal X-ray diffraction experiment, a red transparent crystal of (I) was mounted on the Bruker Smart *APEX* II diffractometer. The experiment was performed at 170 K. The structure was solved by the direct method and refined using *SHELXL-97* program (Sheldrick, 2008). The positions of hydrogen atoms of water molecules were localized from the differential Fourier synthesis and H atoms of the CH_2_ and CH_3_ groups were calculated by the algorithm incorporated in the *SHELXL* program complex. Hydrogen atoms kept fixed with *U*_iso_(H) = 1.5*U*_eq_(C).

### Crystal data

**Table:**

[Co(C_3_H_5_O_2_)_2_(H_2_O)]·H_2_O	*F*(000) = 1000
*M**_r_* = 241.10	*D*_x_ = 1.687 Mg m^−^^3^
Monoclinic, *C*2/*c*	Mo *K*α radiation, λ = 0.71073 Å
Hall symbol: -C 2yc	Cell parameters from 7703 reflections
*a* = 13.997 (4) Å	θ = 2.9–35.9°
*b* = 6.4987 (18) Å	µ = 1.81 mm^−^^1^
*c* = 21.440 (6) Å	*T* = 170 K
β = 103.216 (5)°	Prism, red
*V* = 1898.6 (9) Å^3^	0.5 × 0.3 × 0.2 mm
*Z* = 8	

### Data collection

**Table:**

Bruker APEXII CCD diffractometer	2762 independent reflections
Radiation source: fine-focus sealed tube	2542 reflections with *I* > 2σ(*I*)
graphite	*R*_int_ = 0.032
φ and ω scans	θ_max_ = 30.0°, θ_min_ = 2.0°
Absorption correction: multi-scan (*SADABS*; Bruker, 2007)	*h* = −19→19
*T*_min_ = 0.276, *T*_max_ = 0.332	*k* = −9→9
11986 measured reflections	*l* = −30→30

### Refinement

**Table:**

Refinement on *F*^2^	Primary atom site location: structure-invariant direct methods
Least-squares matrix: full	Secondary atom site location: difference Fourier map
*R*[*F*^2^ > 2σ(*F*^2^)] = 0.026	Hydrogen site location: inferred from neighbouring sites
*wR*(*F*^2^) = 0.067	H atoms treated by a mixture of independent and constrained refinement
*S* = 1.09	*w* = 1/[σ^2^(*F*_o_^2^) + (0.0329*P*)^2^ + 1.4667*P*] where *P* = (*F*_o_^2^ + 2*F*_c_^2^)/3
2762 reflections	(Δ/σ)_max_ < 0.001
130 parameters	Δρ_max_ = 0.41 e Å^−^^3^
5 restraints	Δρ_min_ = −0.57 e Å^−^^3^

### Special details

**Table:**

Geometry. All e.s.d.'s (except the e.s.d. in the dihedral angle between two l.s. planes) are estimated using the full covariance matrix. The cell e.s.d.'s are taken into account individually in the estimation of e.s.d.'s in distances, angles and torsion angles; correlations between e.s.d.'s in cell parameters are only used when they are defined by crystal symmetry. An approximate (isotropic) treatment of cell e.s.d.'s is used for estimating e.s.d.'s involving l.s. planes.
Refinement. Refinement of *F*^2^ against ALL reflections. The weighted *R*- factor *wR* and goodness of fit *S* are based on *F*^2^, conventional *R*-factors *R* are based on *F*, with *F* set to zero for negative *F*^2^. The threshold expression of *F*^2^ > 2σ(*F*^2^) is used only for calculating *R*-factors(gt) *etc*. and is not relevant to the choice of reflections for refinement. *R*-factors based on *F*^2^ are statistically about twice as large as those based on *F*, and *R*- factors based on ALL data will be even larger.

### Fractional atomic coordinates and isotropic or equivalent isotropic displacement parameters (Å^2^)

**Table:**

	*x*	*y*	*z*	*U*_iso_*/*U*_eq_	
Co1	0.248706 (13)	0.18331 (3)	0.244111 (9)	0.01830 (7)	
O1	0.29927 (8)	0.44228 (16)	0.19444 (5)	0.0231 (2)	
H11	0.267 (2)	0.511 (5)	0.1580 (14)	0.080*	
H12	0.362 (2)	0.441 (5)	0.1918 (16)	0.080*	
O2	0.39246 (8)	−0.23440 (17)	0.29662 (6)	0.0262 (2)	
O3	0.23831 (8)	−0.08151 (15)	0.18689 (5)	0.0217 (2)	
O4	0.39309 (8)	0.10838 (17)	0.28586 (5)	0.0246 (2)	
O5	0.47910 (10)	0.4367 (2)	0.17307 (8)	0.0391 (3)	
H51	0.516 (2)	0.328 (5)	0.1935 (15)	0.080*	
H52	0.517 (2)	0.543 (4)	0.1846 (16)	0.080*	
O6	0.21765 (10)	−0.29849 (19)	0.10436 (6)	0.0338 (3)	
C1	0.53359 (12)	−0.0526 (3)	0.35261 (9)	0.0304 (3)	
H1A	0.5732	0.0598	0.3390	0.046*	
H1B	0.5683	−0.1860	0.3505	0.046*	
C2	0.21658 (11)	−0.1222 (2)	0.12565 (7)	0.0229 (3)	
C3	0.1606 (2)	0.0073 (4)	0.01094 (10)	0.0580 (7)	
H3A	0.1450	0.1368	−0.0146	0.087*	
H3B	0.1013	−0.0834	0.0029	0.087*	
H3C	0.2157	−0.0660	−0.0021	0.087*	
C4	0.52401 (19)	−0.0140 (5)	0.42103 (11)	0.0600 (7)	
H4A	0.5908	−0.0101	0.4503	0.090*	
H4B	0.4847	−0.1272	0.4346	0.090*	
H4C	0.4902	0.1206	0.4231	0.090*	
C5	0.43274 (11)	−0.0596 (2)	0.30805 (7)	0.0208 (3)	
C6	0.19027 (18)	0.0594 (3)	0.08116 (9)	0.0418 (5)	
H6A	0.1351	0.1358	0.0932	0.063*	
H6B	0.2480	0.1544	0.0881	0.063*	

### Atomic displacement parameters (Å^2^)

**Table:**

	*U*^11^	*U*^22^	*U*^33^	*U*^12^	*U*^13^	*U*^23^
Co1	0.01681 (10)	0.01335 (10)	0.02365 (11)	0.00019 (7)	0.00237 (7)	0.00019 (7)
O1	0.0216 (5)	0.0199 (5)	0.0281 (5)	0.0003 (4)	0.0061 (4)	0.0025 (4)
O2	0.0192 (5)	0.0173 (5)	0.0387 (6)	−0.0008 (4)	−0.0006 (4)	0.0004 (4)
O3	0.0247 (5)	0.0170 (5)	0.0221 (5)	0.0004 (4)	0.0026 (4)	0.0003 (4)
O4	0.0185 (5)	0.0181 (5)	0.0347 (6)	0.0001 (4)	0.0010 (4)	0.0004 (4)
O5	0.0241 (6)	0.0228 (6)	0.0687 (10)	0.0001 (5)	0.0068 (6)	0.0011 (6)
O6	0.0486 (8)	0.0254 (6)	0.0255 (5)	0.0056 (5)	0.0044 (5)	−0.0032 (4)
C1	0.0188 (7)	0.0231 (8)	0.0443 (9)	0.0001 (6)	−0.0034 (6)	0.0012 (6)
C2	0.0225 (7)	0.0225 (7)	0.0234 (6)	0.0027 (6)	0.0043 (5)	0.0012 (5)
C3	0.089 (2)	0.0507 (13)	0.0271 (9)	0.0158 (13)	−0.0012 (10)	0.0075 (9)
C4	0.0477 (13)	0.0883 (19)	0.0351 (10)	−0.0161 (13)	−0.0093 (9)	0.0042 (12)
C5	0.0170 (6)	0.0199 (7)	0.0253 (6)	0.0002 (5)	0.0044 (5)	−0.0005 (5)
C6	0.0667 (14)	0.0300 (9)	0.0270 (8)	0.0139 (9)	0.0069 (8)	0.0065 (7)

### Geometric parameters (Å, °)

**Table:**

Co1—O2^i^	2.0406 (12)	C1—C5	1.514 (2)
Co1—O4	2.0726 (12)	C1—C4	1.524 (3)
Co1—O3	2.0991 (11)	C1—H1A	1.0000
Co1—O3^i^	2.1058 (11)	C1—H1B	1.0000
Co1—O1	2.1936 (12)	C2—C6	1.509 (2)
Co1—O1^ii^	2.2460 (12)	C3—C6	1.506 (3)
O1—H11	0.92 (3)	C3—H3A	1.0000
O1—H12	0.89 (3)	C3—H3B	1.0000
O2—C5	1.2669 (18)	C3—H3C	1.0000
O3—C2	1.3053 (18)	C4—H4A	1.0000
O4—C5	1.2670 (18)	C4—H4B	1.0000
O5—H51	0.92 (3)	C4—H4C	1.0000
O5—H52	0.87 (3)	C6—H6A	1.0000
O6—C2	1.2346 (19)	C6—H6B	1.0000
			
O2^i^—Co1—O4	178.40 (4)	C4—C1—H1A	109.7
O2^i^—Co1—O3	91.94 (5)	C5—C1—H1B	109.7
O4—Co1—O3	89.44 (4)	C4—C1—H1B	109.7
O2^i^—Co1—O3^i^	91.72 (5)	H1A—C1—H1B	108.2
O4—Co1—O3^i^	87.03 (4)	O6—C2—O3	122.70 (14)
O3—Co1—O3^i^	171.46 (4)	O6—C2—C6	120.92 (14)
O2^i^—Co1—O1	88.83 (5)	O3—C2—C6	116.38 (14)
O4—Co1—O1	89.94 (5)	C6—C3—H3A	109.5
O3—Co1—O1	109.40 (5)	C6—C3—H3B	109.5
O3^i^—Co1—O1	78.38 (5)	H3A—C3—H3B	109.5
O2^i^—Co1—O1^ii^	92.50 (5)	C6—C3—H3C	109.5
O4—Co1—O1^ii^	88.60 (5)	H3A—C3—H3C	109.5
O3—Co1—O1^ii^	77.35 (4)	H3B—C3—H3C	109.5
O3^i^—Co1—O1^ii^	94.79 (5)	C1—C4—H4A	109.5
O1—Co1—O1^ii^	173.08 (3)	C1—C4—H4B	109.5
Co1—O1—Co1^i^	94.44 (5)	H4A—C4—H4B	109.5
Co1—O1—H11	129 (2)	C1—C4—H4C	109.5
Co1^i^—O1—H11	90 (2)	H4A—C4—H4C	109.5
Co1—O1—H12	117 (2)	H4B—C4—H4C	109.5
Co1^i^—O1—H12	118 (2)	O2—C5—O4	124.23 (14)
H11—O1—H12	104 (3)	O2—C5—C1	117.31 (13)
C5—O2—Co1^ii^	131.47 (10)	O4—C5—C1	118.44 (13)
C2—O3—Co1	136.31 (10)	C3—C6—C2	115.31 (17)
C2—O3—Co1^ii^	121.50 (10)	C3—C6—H6A	108.4
Co1—O3—Co1^ii^	101.60 (5)	C2—C6—H6A	108.4
C5—O4—Co1	131.66 (10)	C3—C6—H6B	108.4
H51—O5—H52	104 (3)	C2—C6—H6B	108.4
C5—C1—C4	109.75 (16)	H6A—C6—H6B	107.5
C5—C1—H1A	109.7		

Symmetry codes: (i) −*x*+1/2, *y*+1/2, −*z*+1/2; (ii) −*x*+1/2, *y*−1/2, −*z*+1/2.

### Hydrogen-bond geometry (Å, °)

**Table:**

*D*—H···*A*	*D*—H	H···*A*	*D*···*A*	*D*—H···*A*
O1—H11···O6^iii^	0.92 (3)	1.72 (3)	2.620 (2)	163 (3)
O1—H12···O5	0.89 (3)	1.78 (3)	2.660 (2)	171 (3)
O5—H51···O4^iv^	0.92 (3)	1.90 (3)	2.794 (2)	163 (3)
O5—H52···O2^v^	0.87 (3)	1.91 (3)	2.773 (2)	174 (3)

Symmetry codes: (iii) *x*, *y*+1, *z*; (iv) −*x*+1, *y*, −*z*+1/2; (v) −*x*+1, *y*+1, −*z*+1/2.
